# CSEO – the Cigarette Smoke Exposure Ontology

**DOI:** 10.1186/2041-1480-5-31

**Published:** 2014-07-10

**Authors:** Erfan Younesi, Sam Ansari, Michaela Guendel, Shiva Ahmadi, Chris Coggins, Julia Hoeng, Martin Hofmann-Apitius, Manuel C Peitsch

**Affiliations:** 1 Fraunhofer Institute for Algorithms and Scientific Computing SCAI, Schloss Birlinghoven, 53754 Sankt Augustin, Germany; 2 Philip Morris International R&D, Philip Morris Products S.A., Quai Jeanrenaud 5, 2000 Neuchâtel, Switzerland; 3 Carson Watts Consulting, 1266 Carson Watts Rd, King, NC 27021-7453, USA

**Keywords:** Exposure, Cigarette smoke, Environmental risk, Ontology, Knowledge representation

## Abstract

**Background:**

In the past years, significant progress has been made to develop and use experimental settings for extensive data collection on tobacco smoke exposure and tobacco smoke exposure-associated diseases. Due to the growing number of such data, there is a need for domain-specific standard ontologies to facilitate the integration of tobacco exposure data.

**Results:**

The CSEO (version 1.0) is composed of 20091 concepts. The ontology in its current form is able to capture a wide range of cigarette smoke exposure concepts within the knowledge domain of exposure science with a reasonable sensitivity and specificity. Moreover, it showed a promising performance when used to answer domain expert questions. The CSEO complies with standard upper-level ontologies and is freely accessible to the scientific community through a dedicated wiki at https://publicwiki-01.fraunhofer.de/CSEO-Wiki/index.php/Main_Page.

**Conclusions:**

The CSEO has potential to become a widely used standard within the academic and industrial community. Mainly because of the emerging need of systems toxicology to controlled vocabularies and also the lack of suitable ontologies for this domain, the CSEO prepares the ground for integrative systems-based research in the exposure science.

## Background

Recently, there has been an increased focus in systems toxicology on systems-oriented methodologies that emphasize the understanding on the biological impact of chemical exposures with increased mechanistic granularity [1,2]. In particular, a recent report by the US National Research Council Committee on Toxicity Testing and Assessment of Environmental Agents advocates for a shift away from toxicological assessment at the level of apical endpoints towards the understanding of the effects of an exposure on toxicity pathways [3]. Moreover, the Food and Drug Administration (FDA) recently describes a system-based omics-approach to discover pulmonary biomarkers and to improve the evaluation of tobacco products [4]. This indicates a growing recognition that exposure science should be considered as an integrated part of a systematic approach for risk assessment [5].

To assess biological responses to environmental exposure, a systems-based approach attempts to apply an integrative strategy. A systems-based approach integrates a continuous model from the starting point of exposure to disease outcome [6]. A typical limitation in systems approaches is the lack of standards for harmonization of heterogeneous data types that are experimentally obtained from different resources. Such data types often have various structures, formats and annotations, which adversely affect the degrees of their interoperability and flexibility for integrative methods. Standard terminologies and proper contextual information are necessary for data sharing, reuse, and integration [7]. Recently, biomedical ontologies have emerged in support of systems approaches by facilitating the annotation of bio-simulation models and flexible access to knowledge [8]. The main purpose of ontologies is to organize data and information of a particular knowledge domain in a structured, controlled, and standard manner. Thus the data can be shared among scientists in different research areas or accessed and interpreted using different computational tools. The core of any ontology is a controlled vocabulary that attempts to describe a unified definition for all terms and concepts in a particular subject area [9]. A good example is the Gene Ontology (GO) that provides a controlled vocabulary describing the roles of genes and their products in various organisms [10].

At the heart of systems toxicology is the understanding of signaling pathways perturbed by biologically active substances and the identification of those that have the potential to cause adverse health effects in humans. This requires integrating OMICs data with in vitro and in vivo toxicological endpoints. The goal of systems toxicology is therefore to link disease susceptibility at the molecular level to environmental stress or toxicant effect at the clinical level. Despite advances in various aspects of toxicogenomics, semantic representation of toxicological data and endpoints is still in its infancy. A variety of tools, platforms, and workflows coexist but each uses its own set of terms and ontologies, a challenge for data exchange. Hardy et al. [11] in their review provide an overview of existing toxicology vocabularies and ontologies that are currently being used in predictive toxicology initiatives and applications [11].

Recently, the toxicology OpenTox ontology has been developed to support standard representation of relations between chemical and toxicological datasets and experiments by unified terms. It is part of the OpenTox framework, which aims at unifying access to toxicity data, predictive networks, and validation procedures [12]. One of the advantages of the OpenTox ontology is the combination of several related ontologies that cover common information for chemical compounds, chemical datasets, algorithms, models, assays, in vivo studies, and toxicological endpoints. Moreover, when integrated in a semantic environment, the OpenTox ontology service facilitates registering new resources, remote access, and searching datasets using SPARQL. However, the OpenTox remains a high-level ontology and does not include concept granularity for the majority of its components in particular for the domain of environmental exposure.

Lately, the exposure ontology (ExO) has been proposed to provide the missing link between exposure science and various environmental health disciplines, including toxicology [13]. The main advantage of the ExO is that it provides the first semantic template for representation of exposure information around the following four root concepts: exposure stressor, exposure receptor, exposure event, and exposure outcome. Although the current version of the ExO includes very general and high-level concepts to cover the breadth of the exposure knowledge domain, it still lacks sufficient granularity that is required to capture detailed information. Besides, the ExO is not compliant with the proposed upper-level ontology standards such as the Basic Formal Ontology (BFO) [14] or the Descriptive Ontology for Linguistic and Cognitive Engineering (DOLCE) [15], which makes its integration with existing or new ontologies semantically more difficult. Furthermore, Thomas et al. [16] describe the use of a Smoking Behavior Risk Ontology (SBRO) to represent risk models for phenotypes associated to tobacco smoking behavior [16]. However, the scope of their ontology is limited to nicotine pharmacokinetics, pharmacodynamics, nicotine dependence, and clinical smoking cessation outcomes.

Exposure to tobacco smoke is considered an environmental risk factor to human health and it is involved in the initiation and progression of several respiratory diseases including chronic obstructive pulmonary diseases (COPD) and lung cancer [17,18]. Elimination or minimization of exposure to cigarette smoke provides a clear opportunity to prevent related diseases. Although experiments that measure exposure to environmental tobacco smoke follow – to a large extent – the typical protocols used in toxicology experimental settings, no semantic framework capturing information specific to the domain of cigarette smoke exposure risk is available.

In response to the need for semantic representation of the environmental exposure knowledge domain with particular focus on the cigarette smoke exposure risk, the Cigarette Smoke Exposure Ontology (CSEO) was developed.

## Results

### Purpose of the cigarette smoke exposure ontology

**Figure 1:**
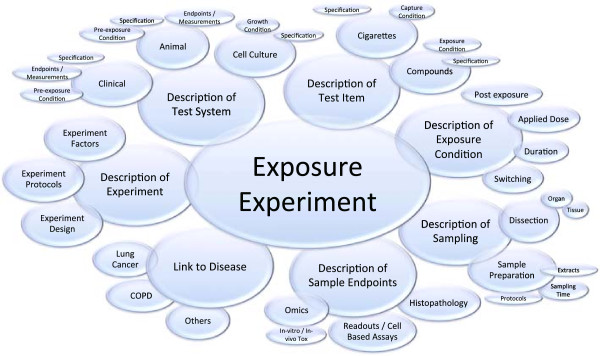
**High-level schematic representation of the CSEO scope.** The scope of CSEO was designed around the key concept of exposure experiment and its substantial elements.

The development of an ontology starts by defining its domain and scope. The scope of the CSEO was defined based on the potential application of the ontology in the domain of environmental exposure and was focused on exposure to cigarette smoke. Since setting a proper scope helps draw boundaries to the knowledge domain included in the ontology, the CSEO is intended to include all concepts and terms that represent processes and elements involved in conducting cigarette smoke exposure experiments, in association with cigarette-smoke related diseases (Figure 1).

The scope of the ontology revolves around the ‘exposure experiment’ concept and covers description of sampling and experimental factors, test items, test systems, exposure condition, and link to diseases. These are the main concepts to be included in the CSEO by following the life cycle of ontology building, as described in the Methods section. Axiomatisation of concepts in the CSEO is based on the axioms provided in the BFO and ExO. For example, the description of an exposure follows the lines of the “exposure event” class in the ExO. We have, furthermore, enriched the ExO classes with extra classes that make the ontology more specific to cigarette smoke rather than just to exposures in general. The reason for choosing these concepts is that they represent the major players in systems toxicology studies conducted in the domain of smoke exposure. Most exposure experiments follow a similar routine summarized as follows: the design, factors, and protocols of an experiment must be defined before conducting the experiment. This is often the case for exploratory systems-based approaches and lesser the case for validated assays. The two main components of an experiment are often a test system and test item, where the test system describes the exposure receptor (e.g., a clinical, in vivo, or in vitro setup), and the test item describes the exposure stressor (e.g., chemical compounds, cigarette smoke, and its characterization). Both of these components require terms that clearly specify the items. These two components interact in an exposure experiment and their interaction is described by the exposure conditions, for example, exposure transport path, frequency, and doses. The exposure condition, therefore, connects the test system and the test items under the experiment description. The exposed test system itself includes sampling procedures, which are bound to various endpoint measurements. In the case of systems-based approaches, the sampling procedures cover a large number of procedures. The sampling of the test items together with the endpoint measurements leads to an outcome, which may be associated with respiratory system diseases.

The main purpose of the ontology is to support annotation of experimental data sets such as the details of the experiment and its design, description of test item, test system, as well as the exposure path to outcomes. Additional file 1 shows an example on the use of CSEO to annotate experiments. GeneChip Microarray experiments generate high-throughput transcriptomic data that can be reused for other research topics than the originally designed experiment. Therefore, the FGED (Functional Genomics Data) society created standards to exchange these and other similar data types related to functional genomics. These standards not only include the format of exchange but also the minimum requirements for experimental annotation so that experimental data can be correctly reproduced and reused. The exchange file format is called MAGE-TAB [19], which includes an IDF file for the definition of the investigation, a SDRF file for the specification of each sample, and an ADF file for the specification of the microarray analyte layout. This file format is supported by the repository ArrayExpress [20] and gives open access to a large number of functional genomics datasets.

While MAGE-TAB defines the exchange format, there is another standard that describes the required annotation level, MIAME [21] the Minimum Information About a Microarray Experiment. Additional file 1 shows an example of the SDRF file that is MAGE-TAB and MIAME compliant. Each row indicates the biological samples with annotations and protocols for biological sample transformation. The data model starts with a subject, which is an animal model including additional information about type, strain, and gender. When a protocol applies, the biomaterial is changed, here from an untreated animal to a treated animal. The treatment is further described with the exposure item, brand, smoking regimen, nicotine concentration, exposure path, and exposure duration. The next protocol defines a post-exposure treatment and affects only part of the samples. After all exposures, the animal is dissected into organ parts that are described by the next protocol. The organ part is now further defined as frozen alveolar tissue area from left lung of each animal. The next protocols define lysis in this tissue and the extraction of RNA that is hybridized on a GeneChip. The SDRF file ends with the reference to the raw data file names, processed data file name, and a summary of all experimental factor values. All protocols are defined in the IDF file (not shown). MAGE-TAB requires the use of ontology defined terms. The ontology resource is specified with location and version in the IDF. Yellow marked columns in Additional file 1 show the CSEO annotations that cover a large fraction of the SDRF file and ensure rich and proper annotation. The annotation level of this file is much richer than the MIAME requirement and supports the reproducibility and reusability of experimental data.

Furthermore, conceptualizing and organizing this knowledge domain in the form of an ontology allows efficient augmentation of biological knowledge retrieval and extraction. Therefore, the sensitivity to which biological mechanisms are modulated in response to different risk factors posed by smoking toxicants in the lungs can be captured.

### Framework and architecture of the CSEO

**Figure 2:**
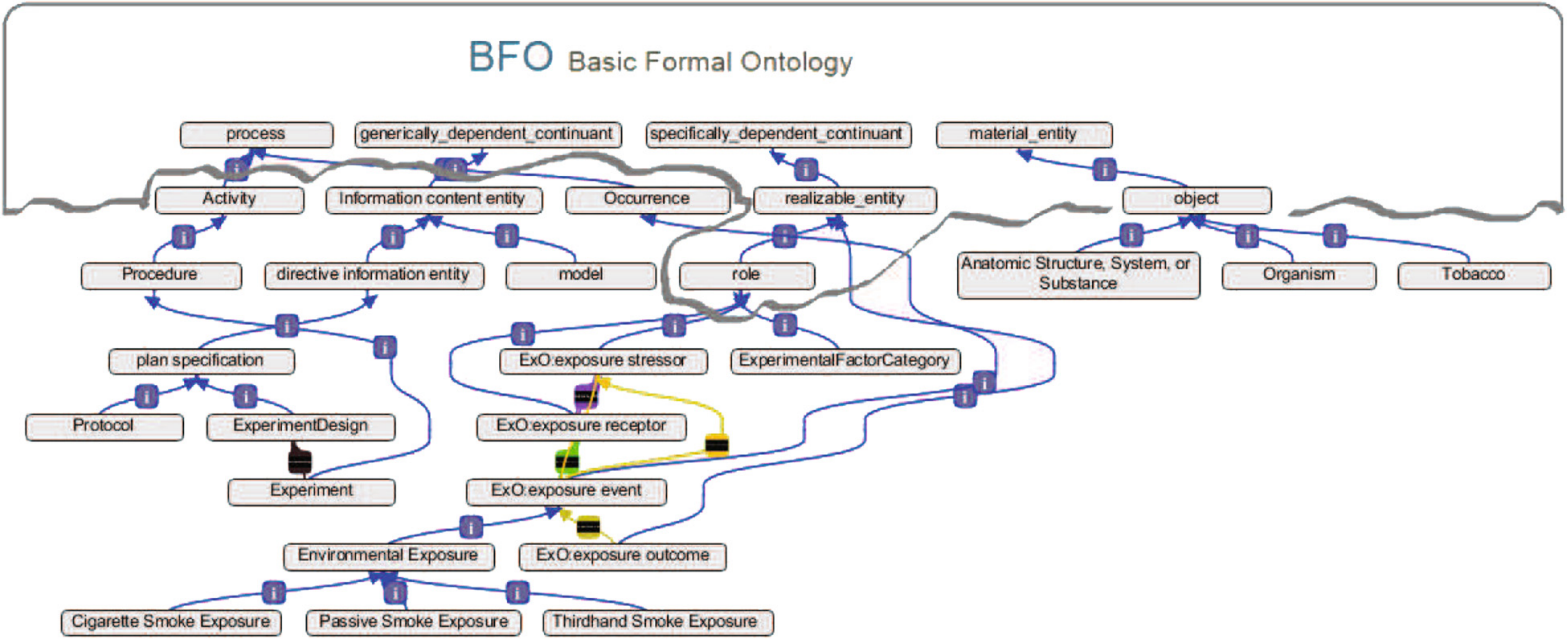
**Schematic representation of the main ontology classes and class provenance between BFO, ExO, and CSEO.** Arrow legend: blue: is-a relations; yellow: ExO: is_associated_with; orange: ExO: interacts_with_an_exposure_stressor_via; brown: MGED: has_experiment_ design; violet: ExO: interacts_with.

The CSEO was designed to be compliant with the Basic Formal Ontology (BFO). The BFO was adopted to define the upper-level standard architecture. The BFO is designed to support development of domain ontologies for scientific research [22]. On the other hand, the ExO is the only existing and intuitive semantic framework used by the exposure science community that provides a good template for plugging in subdomain ontologies related to the exposure domain. Therefore, the ExO superclasses were used as root concepts for the CSEO. Accordingly, the CSEO populates the ExO for the concepts of the cigarette smoke risk subdomain and also complies with requirements of the OBO Foundry and RO (Relation Ontology). Figure 2 depicts the architecture of the CSEO in relation to BFO and ExO and its main classes. Such an architecture is expected to incorporate provenance into the CSEO so that concepts can be traced back to their corresponding upper-level classes in ExO and BFO.

The CSEO comes in two different versions: the main CSEO version is a BFO-compliant ontology, and the second version is a controlled vocabulary version, hereafter referred to as “lexical version”. The CSEO-BFO version consists of the BFO top-level hierarchy into which the adjusted ExO hierarchy was plugged. The CSEO classes were organized underneath these layers as a third layer of granularity. This is the so-called “computer-readable” format of the CSEO, which represents the formal ontology. The lexical version, on the other hand, forms the so-called “expert-readable” format and does not claim to be a standard-adhering ontology in itself. Instead, it is an access point to the CSEO classes that is intuitive and easy to navigate for medical and biological experts. This lexical version supports the creation and review of the ontology by various experts within the field. It, furthermore, creates a categorization of ontology classes and terms into ‘context categories’ inside the knowledge domain. This is usable also for context-sensitive text mining i.e., it contains a branch that collects all terms related to exposure outcomes (including terms which are not necessarily exposure types) compared to the CSEO-BFO version where they have to be collected manually. Both versions are available on the CSEO dedicated wiki website.

### Three-dimensional evaluation of the CSEO

#### *Structural measure*

Measurement of the structural dimension of the ontology reflects the organizational patterns of the concepts in the ontology. The first draft of CSEO (version 1.0) is composed of 20091 concepts, including the BFO and ExO classes. Additional file 2 provides several metrics on structural properties of the ontology. These metrics include ‘breadth’, which relates to the cardinality of paths; ‘depth’, which relates to the cardinality of paths in a graph; ‘tangledness’, which relates to multi-hierarchical nodes; and ‘fanout factor’, which relates to the dispersion of nodes.

As shown in Additional file 2, the high number of classes and leaves together with high values for average width and the fanout factor, point towards a broad coverage of concepts by the ontology whereas the values for depth show specificity of the concept types to the domain of cigarette smoke exposure risk. The tangledness factor of 0.71 indicates the presence of multi-hierarchical nodes in the ontology (i.e. categories having multiple parents). This is beneficial when greater crosslinking of the domain concepts is desired. Different relation types from RO were used to relate concepts in the CSEO including ‘part_of’, ‘precedes’, ‘has_participant’, etc. Figure 2 illustrates the relational view of the second-level concepts in the CSEO.

#### *Functional measure*

Measuring the functional dimension of the ontology indicates how well the conceptualization of the ontology captures the semantic space of the knowledge domain. The lexicalized ontology was used to calculate precision, recall, and F-score values (69.23, 77.81, 73.26, respectively).

The result of this evaluation shows that the ontology in its current form is able to capture a wide range of concepts related to cigarette smoke exposure in the knowledge domain of exposure with a reasonable sensitivity and specificity towards manual curation. The F-score of above 73% reflects the quality output of the ontological search in the published knowledge domain of cigarette smoke exposure risk.

#### *Usability profile*

**Figure 3:**
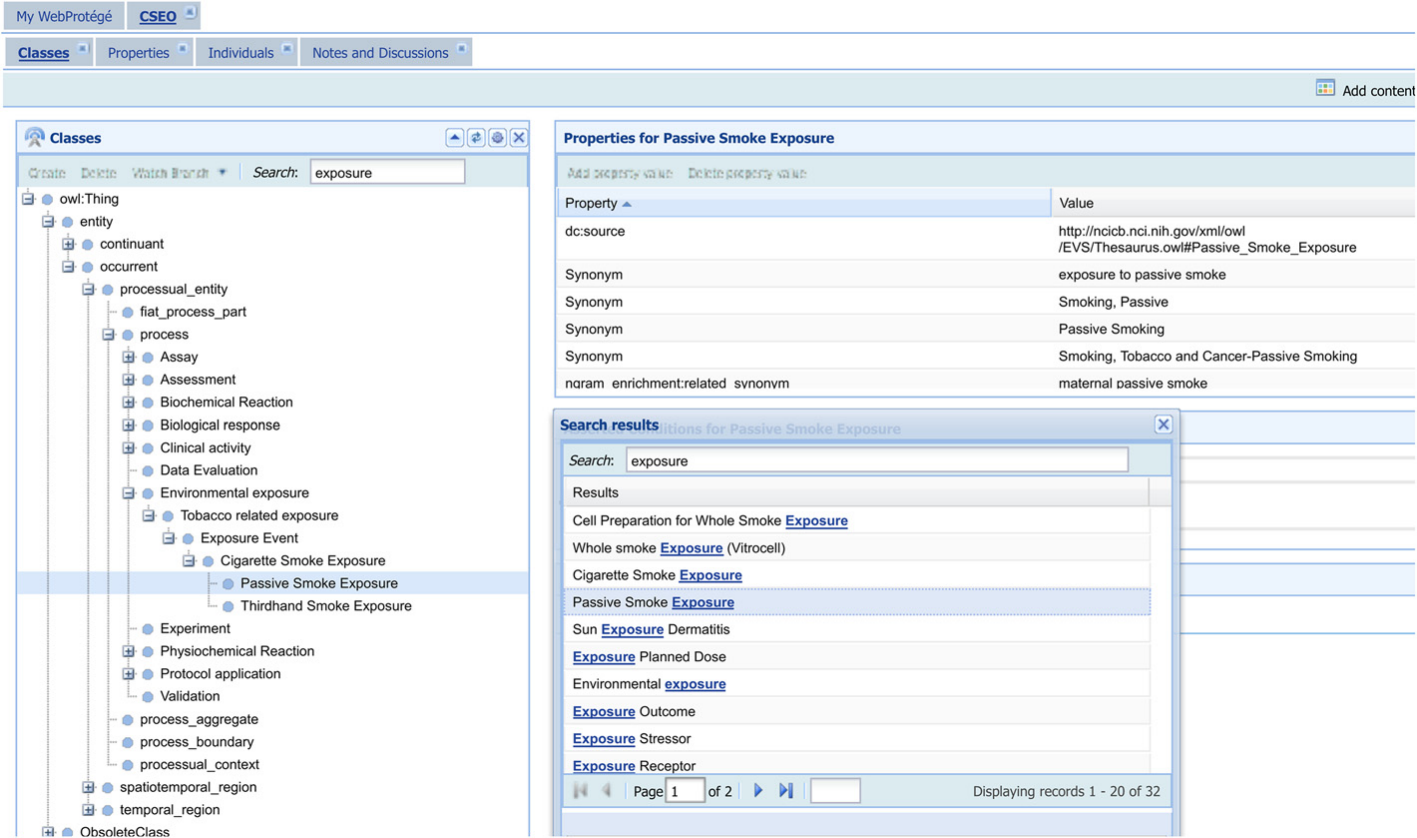
Illustration of term search and navigation through the CSEO.

Usability profile of an ontology is defined by the extent of user-friendliness of the ontology in terms of easy navigation, knowledge accessibility, and meta-information availability. Navigation of the CSEO and its user interface has been facilitated using the WebProtégé software, which provides a web-based access to the content of the ontology without the need for software installation [23]. By following the hyperlink provided on the wiki website under “CSEO access”, the user is directed to the WebProtégé page in which clicking CSEO launches the formal BFO-compliant ontology whereas clicking CSEO-Expert Readable hyperlink launches the hierarchy of controlled vocabulary underlying CSEO. The search field makes it possible to search for any CSEO-related concept and locate it in the tree (Figure 3). Feedbacks can be provided through the same portal and a dedicated team will process them.

To increase the level of efficiency in accessing different views (subdomains) of the ontology, the ExO root concepts were used for further classification of the CSEO instants. By this means, tracking exposure-specific concepts for users becomes easier and more efficient. Meta-information (i.e. annotations including synonyms, definition, and reference) is provided for each concept in the CSEO to enable users accessing relevant information.

Since a proper documentation is needed to ensure direct access and efficient usability of the ontology, a wiki environment was created that contains instructions for using the ontology, documentation on purpose and scope of the ontology, and information about interfacing to the ontology. The wiki is accessible through the following hyperlink in FireFox and Safari browsers: https://publicwiki-01.fraunhofer.de/CSEO-Wiki/index.php/Main_Page.

### Use-case scenario: answering competency questions by experts

Ontology-driven information retrieval and extraction systems will guide analysis of literature in precisely answering complex scientific questions [24]. The lexicalized form of the CSEO was used to automatically retrieve and extract domain specific knowledge related to cigarette smoke exposure risk from PubMed abstracts (see Methods). Experts in the knowledge domain of cigarette smoke exposure risk were asked to design several complex questions to be posed to the ontology. The following questions were considered to test the performance of the ontology:

– What are the potential effects of the toxicity induced by tobacco smoke constituents on smokers?

– Which toxicological studies are available that measure total particulate matter in electrically heated cigarettes?

– Which documents report on the use of experimental mouse models for investigating the effect of cigarette smoke exposure on the risk of COPD?

Queries were formulated in the SCAIView environment using the CSEO terminology. SCAIView displays named entities by markup of the text (e.g. PubMed abstracts). The key feature of SCAIView is the possibility to perform ontological search in biomedical text using concept hierarchies and synonyms associated with each concept in the ontology. While using the ontology in SCAIView, the hierarchical organization of the ontology was preserved by transforming the ontology OWL file into an XML tree structure. Subsequently, retrieved documents were manually checked for containing correct answers to the posed competency questions. Table 1 summarizes these queries, their corresponding retrieval rate, and reference to the relevant documents that contain correct answers to competency questions. Titles of both relevant and irrelevant abstracts are listed in Additional file 3.

**Table 1:** Answering competency questions using CSEO-driven semantic search in PubMed abstracts

**Query (22.03.2013)**	**No. of retrieved docs:**	**No. of relevant docs:**	**PMIDs of relevant documents:**
(([CSEO: “Smoke Constituent”]) AND [CSEO:“ Toxicity”]) AND [CSEO: “Tobacco”]	21	17 (80.95%)	14521141 [25], 1188959 [26], 18848577 [27], 21651432 [28], 17661226 [29], 2002748 [30], 12857635 [31], 19330121 [32], 14698566 [33], 11731039 [34], 18383128 [35], 16859820 [36], 21651433 [37], 21417965 [38], 2165143 1[39], 15072838 [40] , 18464053 [41]
([CSEO: “Electrically heated cigarette”]) AND [CSEO: “Total Particulate Matter”]	7	7 (100%)	12975773 [42], 12975774 [43], 14698566 [33], 12975771 [44], 18590791 [45], 12975772 [46], 16963170 [47]
(([CSEO: “Mouse model”]) AND [CSEO: “Cigarette Smoke Exposure”]) AND [MeSH Disease: “Pulmonary Disease Chronic Obstructive”]	9	9 (100%)	20133926 [48], 19017996 [49], 23044435 [50], 22279084 [51], 18988919 [52], 21700603 [53], 20228194 [54], 19491340 [55],16510458 [56]

**Figure 4:**
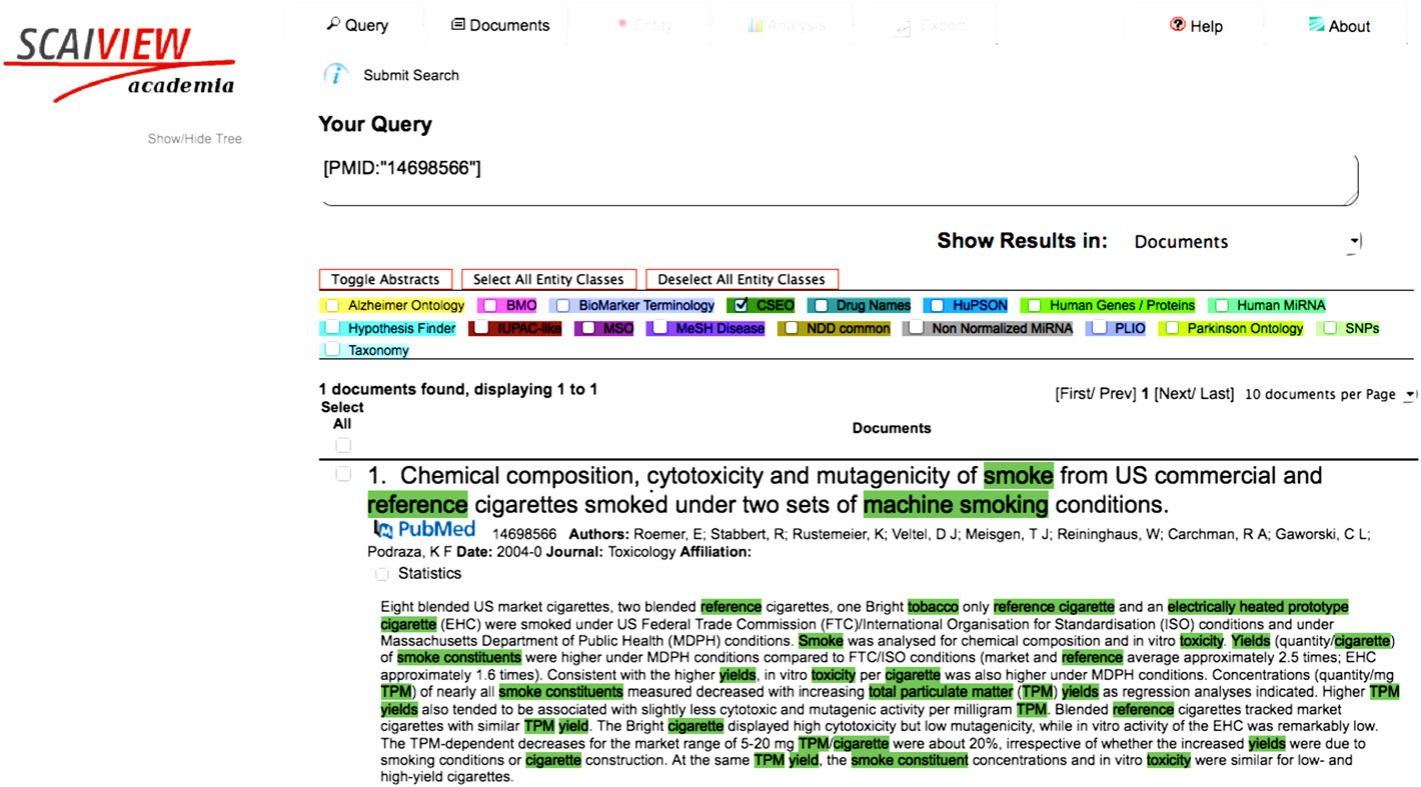
**An example of highlighted CSEO terms in the PubMed abstracts as appears in the SCAIView environment.** The highlighted terms guide users to informative statements and facilitates their detection, quality check and extraction.

These results indicate that application of the CSEO-derived terminology to the semantic literature search leads to retrieval of highly relevant publications containing the correct answer to the posed competency question. Moreover, highlighted CSEO concepts (terms) by SCAIView allow users to detect and extract knowledge statements, as illustrated in Figure 4. The CSEO terminology can be accessed through the SCAIView search engine under: http://www.scaiview.com/scaiview-academia.html.

## Discussion

The CSEO covers relevant concepts in the field of systems-based toxicology assessment and includes many terms from the conventional toxicology assessment. Thus, the CSEO enables users to capture and integrate exposure information from the beginning of the experiment to the point of outcome measurement. Compared to other relevant ontologies, the CSEO covers a large number of concept classes including the 44 external ontologies. Additionally, the CSEO uses semi-automated methods for the term extraction and evaluation and therefore ensures good coverage of the knowledge domain.

Another advantage of the CSEO over the existing related ontologies is the enrichment of high-resolution concepts that extends the higher-level exposure ontology in areas where existing ontologies are particularly weak. For instance, the CSEO describes mouse and rat strains that are commonly used in exposure experiments, includes human anatomy with a dedicated subclass to microanatomy of the respiratory system, and articulates staging of progressive diseases. Moreover, the CSEO can be used for text mining and knowledge discovery purposes because the CSEO is a lexicalized ontology that supports ontology-driven information retrieval and extraction as described in the application scenario. Finally, the ability to use the CSEO in different systems may be facilitated by the BFO upper-level ontology. Thus, various subontologies relevant to exposure can be integrated with the ExO-CSEO structure under the BFO framework.

Similar to other ontologies, the CSEO suffers from the sparse granularity and misclassification of concepts in some parts of the ontology. Other shortcomings common to all ontologies such as missing concepts, lack of standard definitions, and incompleteness of synonym lists should be addressed by engagement of the research community and inclusion of their feedback in the process of ontology enrichment. To facilitate the community contribution, a website has been prepared with the aim of collecting users’ feedback and providing access to the latest version of the ontology. With the public release of the ontology, it is hoped to reach out to the broader community and collect feedback and comments, which will be integrated in the future versions of the CSEO and be used to improve the ontology. With the version 1.0 of the CSEO, the ontology is sufficiently established to be useful for the scientific community. Furthermore, the project team will continue to review articles, abstracts, and other resources relevant for the domain and to extract novel terms and synonyms. New releases of the CSEO will be announced and made available through the NCBO’s bioportal.

## Conclusions

With the creation of the CSEO including relevant terms for describing exposure experiments, it can serve as a powerful glossary for definition finding and relationship visualization, facilitating the right use of terms. The CSEO has the potential to grow in the future and be used as a dictionary for various processes such as controlling internal documents (e.g. Excel Workbooks) or efficient use of Laboratory Information Management Systems (LIMS). This functionality can be used for the identification of relevant information (internally or publicly) or for the extraction of relevant knowledge statements.

## Methods

### Defining scope of the CSEO

To define the scope, a qualitative survey was performed involving various experts in the domain of environmental exposure. Experts in toxicology, molecular biology, and clinical pathology fields in PMI were consulted and asked for their input on the concept classes that they deem as necessary to describe the knowledge domain of environmental exposure from their viewpoint. Based on this input, boundaries of the knowledge domain to be presented by CSEO was determined as depicted in Figure 1.

### Resources and tools

Different resources were used for construction of the ontology (Additional file 4). General and common concepts, for which an established ontological definition exists, were captured. 44 publicly available ontologies listed in Additional file 4 were re-used and the relevant terms/classes/concepts were selectively integrated in the CSEO along with their annotations. Specialized terms were collected from various contributors mainly used for internal process and workflow tracking in systems, such as Laboratory Information Management Systems (LIMS). Literature sources either were searched by keywords (e.g. smoke, toxicity, cigarette, tobacco in PubMed) or were recommended by experts (e.g. CORESTA publications or handbooks). Additionally, relevant publicly available abstracts, a number of relevant full-text articles, as well as “The Handbook of Cigarette Smoke Toxicity” by David Bernhard were reviewed. Here, relevant text bodies were manually annotated, relevant terms were extracted and enriched with synonyms and integrated into the ontology.

The Protégé 4.2 (Build 276) [57], developed and maintained by The National Center for Biomedical Ontology together with its inbuilt HermiT 1.3.3 reasoner [58] were used to construct the ontology. The Knowtator plugin [59] was used for manual annotation of abstracts inside the Protégé environment. The text-mining tool ProMiner [60] was utilized for named entity recognition of ontology terms in PubMed abstracts and results were integrated with SCAIView [61] for context-sensitive visualization of query results.

### Ontology development and evaluation process

During the process of ontology building, a hybrid approach combining both bottom-up and top-down methods was adopted so that the ontology was populated at the level of superclasses and subclasses simultaneously. The development of the CSEO was accomplished in four phases according to the common life cycle of the ontology building [62].

#### *Phase I: Knowledge acquisition and conceptualization*

**Figure 5:**
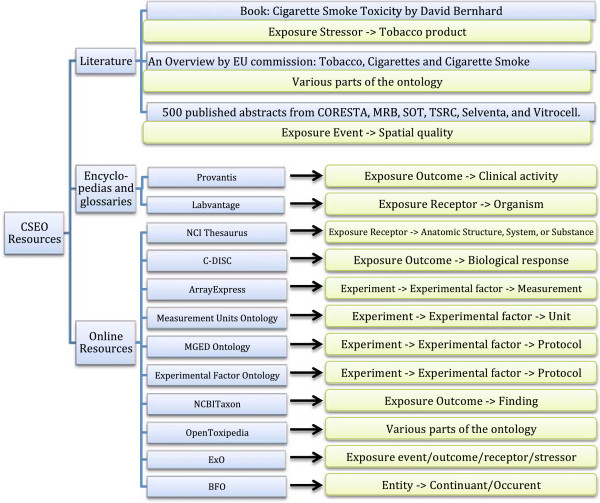
Mapping resources used for generating the ontology contents to their corresponding branches in the CSEO.

Concepts were extracted from previously identified resources (see Additional file 4). Resources were classified into two groups based on their contents: structured content and unstructured content. Concepts from structured contents such as tables, ontologies, and lists were integrated automatically whereas concepts from unstructured contents such as free text of publications were manually inspected and extracted with the help of annotation tools. Figure 5 describes the cardinal mapping of resources to the ontology contents. All concepts in the ontology were annotated by additional information including synonym(s), definition(s), and reference(s). In the BFO version of the CSEO, relationships among concepts were defined based on the standard relation types in the Relation Ontology (RO) [63] and were checked using the HermiT reasoner.

#### *Phase II: Terminology analysis and concept enrichment*

Transformation of the ontology OWL format into a dictionary file was achieved using a Java script. The script extracts concept names and the corresponding synonyms from the ontology OWL structure and assigns unique identifiers to each concept. This dictionary was incorporated into ProMiner for named entity recognition. In a subsequent step, the major super-class concepts were used as keywords for queries in PubMed. Five hundred relevant abstracts were chosen from the result list of each concept search. After compiling all abstracts, the corpus was randomly divided into a training set (250 abstracts) and test set (250 abstracts) using the randomization command in Linux. To create the reference gold standard, suitable annotation guidelines were developed so that annotators are guided to keep the breadth and depth of the ontology in mind. For enrichment purposes (here optimizing both the ontology concepts and the corresponding dictionary), the training set was analyzed for false-negative entities, which — after individual expert evaluation — was added to the ontology. Classes were annotated both manually and automatically by mapping them to external ontologies. For this purpose, the National Center for Biomedical Ontology (NCBO) was used [64]. CSEO classes were manually annotated with equivalent external ontology classes using an annotation property. These annotations were then used to automatically retrieve synonym information via the NCBO services. The evaluation process required the performance comparison between automatically and manually annotated text from the same set.

#### *Phase III: Evaluation*

A metric-based approach evaluating the ontology was used in three dimensions after the completion of the ontology [65]. Structural evaluation was performed by calculating features such as depth, breadth, and other topological features. To evaluate the functional quality of the ontology in terms of measuring the boundaries of the knowledge domain it captures, precision, recall, and F-score values were calculated. Precision is the number of true positives (TP) divided by the sum of TP and false positives (FP). Recall is the number of TP divided by the number of results that should have been returned (true positives (TP) + false negatives (FN)). The F-score = 2 × (precision × recall)/(precision + recall). These values were derived from the longest string match found between automatically annotated words using ProMiner and the human-curated gold standard annotation for each abstract in the selected corpus [66].

#### *Phase IV: Visualization of concepts through the text*

The ontology was integrated into the SCAIView literature mining and visualization environment.

## Competing interests

Authors declare no competing interests.

## Authors’ contributions

EY conceived of the study, carried out ontology construction studies, participated in anntation and evaluation, and drafted the manuscript. SA conceived of the study, carried out data collection, participated in ontology construction and evaluation, and helped to draft the manuscript. MG performed ontology formalization, dictionary generation and technical evaluation. SA performed ontology construction and participated in ontology annotation and evaluation. CC participated in stakeholder engagement. JH participated in the design of the study and coordination. MHA and MCP conceived of the study and participated in its design and coordination. All authors read and approved the final manuscript.

## Supplementary Material

Additional file 1 MAGE-TAB SDRF file with CSEO classes. Click here for file

Additional file 2 CSEO ontology metrics. Click here for file

Additional file 3 **Titles of retrieved PubMed abstracts for answering competency questions in Table** 1**.** Click here for file

Additional file 4 Resources used for construction of CSEO. Click here for file

## References

[B1] Bhattacharya S Zhang Q Carmichael PL Boekelheide K Andersen ME Toxicity testing in the 21 century: defining new risk assessment approaches based on perturbation of intracellular toxicity pathways PLoS One 2011 6 e20887 21701582 10.1371/journal.pone.0020887 PMC3118802

[B2] Keller DA Juberg DR Catlin N Farland WH Hess FG Wolf DC Doerrer NG Identification and characterization of adverse effects in 21st century toxicology Toxicol Sci 2012 126 291 297 22262567 10.1093/toxsci/kfr350 PMC3307604

[B3] Krewski D Acosta D Jr Andersen M Anderson H Bailar JC 3rd Boekelheide K Brent R Charnley G Cheung VG Green S Jr Kelsey KT Kerkvliet NI Li AA McCray L Meyer O Patterson RD Pennie W Scala RA Solomon GM Stephens M Yager J Zeise L Toxicity testing in the 21st century: a vision and a strategy J Toxicol Environ Health 2010 13 51 138 10.1080/10937404.2010.483176 PMC4410863 20574894

[B4] Wang H Mattes WB Richter P Mendrick DL An omics strategy for discovering pulmonary biomarkers potentially relevant to the evaluation of tobacco products Biomark Med 2012 6 849 860 23227851 10.2217/bmm.12.78

[B5] Sheldon LS Cohen Hubal EA Exposure as part of a systems approach for assessing risk Environ Health Perspect 2009 117 1181 1194 10.1289/ehp.0800407 PMC2721858 19672394

[B6] Waters MD Fostel JM Toxicogenomics and systems toxicology: aims and prospects Nat Rev Genet 2004 5 936 948 15573125 10.1038/nrg1493

[B7] Sansone SA Rocca-Serra P Field D Maguire E Taylor C Hofmann O Fang H Neumann S Tong W Amaral-Zettler L Begley K Booth T Bougueleret L Burns G Chapman B Clark T Coleman LA Copeland J Das S de Daruvar A de Matos P Dix I Edmunds S Evelo CT Forster MJ Gaudet P Gilbert J Goble C Griffin JL Jacob D Toward interoperable bioscience data Nat Genet 2012 44 121 126 22281772 10.1038/ng.1054 PMC3428019

[B8] Hoehndorf R Dumontier M Gennari JH Wimalaratne S de Bono B Cook DL Gkoutos GV Integrating systems biology models and biomedical ontologies BMC Syst Biol 2011 5 124 21835028 10.1186/1752-0509-5-124 PMC3170340

[B9] Courtot M Juty N Knüpfer C Waltemath D Zhukova A Dräger A Dumontier M Finney A Golebiewski M Hastings J Hoops S Keating S Kell DB Kerrien S Lawson J Lister A Lu J Machne R Mendes P Pocock M Rodriguez N Villeger A Wilkinson DJ Wimalaratne S Laibe C Hucka M Le Novère N Controlled vocabularies and semantics in systems biology Mol Syst Biol 2011 7 543 22027554 10.1038/msb.2011.77 PMC3261705

[B10] Ashburner M Ball CA Blake JA Botstein D Butler H Cherry JM Davis AP Dolinski K Dwight SS Eppig JT Harris MA Hill DP Issel-Tarver L Kasarskis A Lewis S Matese JC Richardson JE Ringwald M Rubin GM Sherlock G Gene ontology: tool for the unification of biology. The Gene Ontology Consortium Nat Genet 2000 25 25 29 10802651 10.1038/75556 PMC3037419

[B11] Hardy B Apic G Carthew P Clark D Cook D Dix I Escher S Hastings J Heard DJ Jeliazkova N Judson P Matis-Mitchell S Mitic D Myatt G Shah I Spjuth O Tcheremenskaia O Toldo L Watson D White A Yang C Toxicology ontology perspectives ALTEX 2012 29 139 156 22562487 10.14573/altex.2012.2.139

[B12] Tcheremenskaia O Benigni R Nikolova I Jeliazkova N Escher SE Batke M Baier T Poroikov V Lagunin A Rautenberg M Hardy B OpenTox predictive toxicology framework: toxicological ontology and semantic media wiki-based OpenToxipedia J Biomed Semant 2012 3 Suppl 1 S7 10.1186/2041-1480-3-S1-S7 PMC3337268 22541598

[B13] Mattingly CJ McKone TE Callahan MA Blake JA Hubal EA Providing the missing link: the exposure science ontology ExO Environ Sci Technol 2012 46 3046 3053 22324457 10.1021/es2033857 PMC3314380

[B14] Vogt L Grobe P Quast B Bartolomaeus T Accommodating ontologies to biological reality - top-level categories of cumulative-constitutively organized material entities PLoS One 2012 7 e30004 22253856 10.1371/journal.pone.0030004 PMC3253816

[B15] Gangemi A Guarino N Masolo C Oltramari A Schneider L Richard Benjamins V Gómez-Pérez A Sweetening Ontologies with DOLCE Proceedings of the 13th International Conference Knowledge Engineering and Knowledge Management (EKAW2002) 2002 Berlin Heidelberg: Springer-Verlag 166 181

[B16] Thomas PD Mi H Swan GE Lerman C Benowitz N Tyndale RF Bergen AW Conti DV Pharmacogenetics of Nicotine Addiction and Treatment Consortium. A systems biology network model for genetic association studies of nicotine addiction and treatment Pharmacogenet Genomics 2009 19 538 551 19525886 10.1097/FPC.0b013e32832e2ced PMC6485245

[B17] Shields PG Molecular epidemiology of lung cancer Ann Oncol 1999 10 Suppl 5 S7 S11 10582132 10.1093/annonc/10.suppl_5.s7

[B18] Celli BR Chronic obstructive pulmonary disease and lung cancer: common pathogenesis, shared clinical challenges Proc Am Thorac Soc 2012 9 74 79 22550249 10.1513/pats.201107-039MS

[B19] Rayner TF Rocca-Serra P Spellman PT Causton HC Farne A Holloway E Irizarry RA Liu J Maier DS Miller M Petersen K Quackenbush J Sherlock G Stoeckert CJ Jr White J Whetzel PL Wymore F Parkinson H Sarkans U Ball CA Brazma A A simple spreadsheet-based, MIAME-supportive format for microarray data: MAGE-TAB BMC Bioinformatics 2006 7 489 17087822 10.1186/1471-2105-7-489 PMC1687205

[B20] Brazma A Parkinson H Sarkans U Shojatalab M Vilo J Abeygunawardena N Holloway E Kapushesky M Kemmeren P Lara GG Oezcimen A Rocca-Serra P Sansone SA ArrayExpress–a public repository for microarray gene expression data at the EBI Nucleic Acids Res 2003 31 68 71 12519949 10.1093/nar/gkg091 PMC165538

[B21] Brazma A Hingamp P Quackenbush J Sherlock G Spellman P Stoeckert C Aach J Ansorge W Ball CA Causton HC Gaasterland T Glenisson P Holstege FC Kim IF Markowitz V Matese JC Parkinson H Robinson A Sarkans U Schulze-Kremer S Stewart J Taylor R Vilo J Vingron M Minimum information about a microarray experiment (MIAME)-toward standards for microarray data Nat Genet 2001 29 365 371 11726920 10.1038/ng1201-365

[B22] Grenon P Smith B Goldberg L Biodynamic ontology: applying BFO in the biomedical domain Stud Health Technol Inform 2004 102 20 38 15853262

[B23] Tudorache T Nyulas CI Noy NF Musen MA WebProtégé: A collaborative ontology editor and knowledge acquisition tool for the web Semant Web 2013 4 89 99 23807872 10.3233/SW-2012-0057 PMC3691821

[B24] Spasic I Ananiadou S McNaught J Kumar A Text mining and ontologies in biomedicine: making sense of raw text Briefings Bioinf 2005 6 239 251 10.1093/bib/6.3.239 16212772

[B25] Rogers JM Abbott BD Screening for developmental toxicity of tobacco smoke constituents Toxicol Sci 2003 75 2 227 228 14521141 10.1093/toxsci/kfg215

[B26] Pilotti A Ancker K Arrhenius E Enzell C Effects of tobacco and tobacco smoke constituents on cell multiplication in vitro Toxicology 1975 5 49 62 1188959 10.1016/0300-483x(75)90069-4

[B27] Stellman SD Djordjevic MV Monitoring the tobacco use epidemic II: The agent: Current and emerging tobacco products Prev Med 2009 48 Suppl 1 S11 S15 18848577 10.1016/j.ypmed.2008.09.004 PMC2667905

[B28] Coggins CR Liu J Merski JA Werley MS Oldham MJ A comprehensive evaluation of the toxicology of cigarette ingredients: aliphatic and aromatic carboxylic acids Inhal Toxicol 2011 1 119 140 21651432 10.3109/08958378.2010.549528

[B29] Walaszek Z Hanausek M Slaga TJ The role of skin painting in predicting lung cancer Int J Toxicol 2007 26 345 351 17661226 10.1080/10915810701490422

[B30] Carr LA Basham JK Effects of tobacco smoke constituents on MPTP-induced toxicity and monoamine oxidase activity in the mouse brain Life Sci 1991 48 1173 1177 2002748 10.1016/0024-3205(91)90455-k

[B31] Stavanja MS Ayres PH Meckley DR Bombick BR Pence DH Borgerding MF Morton MJ Mosberg AT Swauger JE Toxicological evaluation of honey as an ingredient added to cigarette tobacco J Toxicol Environ Health 2003 66 1453 1473 10.1080/15287390306413 12857635

[B32] Brown BG Borschke AJ Doolittle DJ An analysis of the role of tobacco-specific nitrosamines in the carcinogenicity of tobacco smoke Nonlinearity Biol Toxicol Med 2003 1 179 198 19330121 10.1080/15401420391434324 PMC2651603

[B33] Roemer E Stabbert R Rustemeier K Veltel DJ Meisgen TJ Reininghaus W Carchman RA Gaworski CL Podraza KF Chemical composition, cytotoxicity and mutagenicity of smoke from US commercial and reference cigarettes smoked under two sets of machine smoking conditions Toxicology 2004 195 31 52 14698566 10.1016/j.tox.2003.08.006

[B34] Rustemeier K Stabbert R Haussmann HJ Roemer E Carmines EL Evaluation of the potential effects of ingredients added to cigarettes. Part 2: chemical composition of mainstream smoke Food Chem Toxicol 2002 40 93 104 11731039 10.1016/s0278-6915(01)00085-0

[B35] Talbot P In vitro assessment of reproductive toxicity of tobacco smoke and its constituents Birth Defects Res C Embryo Today 2008 84 61 72 18383128 10.1002/bdrc.20120

[B36] Baker RR The generation of formaldehyde in cigarettes–Overview and recent experiments Food Chem Toxicol 2006 44 1799 1822 16859820 10.1016/j.fct.2006.05.017

[B37] Coggins CR Frost-Pineda K Smith DC Oldham MJ A comprehensive evaluation of the toxicology of cigarette ingredients: aromatic and aliphatic alcohol compounds Inhal Toxicol 2011 1 141 156 21651433 10.3109/08958378.2010.551552

[B38] Gaworski CL Oldham MJ Wagner KA Coggins CR Patskan GJ An evaluation of the toxicity of 95 ingredients added individually to experimental cigarettes: approach and methods Inhal Toxicol 2011 1 1 12 21417965 10.3109/08958378.2010.543187

[B39] Coggins CR Jerome AM Edmiston JS Oldham MJ A comprehensive evaluation of the toxicology of cigarette ingredients: aliphatic carbonyl compounds Inhal Toxicol 2011 1 102 118 21651431 10.3109/08958378.2010.545842

[B40] Baker RR Massey ED Smith G An overview of the effects of tobacco ingredients on smoke chemistry and toxicity Food Chem Toxicol 2004 42 Suppl S53 S83 15072838 10.1016/j.fct.2004.01.001

[B41] Moennikes O Vanscheeuwijck PM Friedrichs B Anskeit E Patskan GJ Reduced toxicological activity of cigarette smoke by the addition of ammonia magnesium phosphate to the paper of an electrically heated cigarette: subchronic inhalation toxicology Inhal Toxicol 2008 20 647 663 18464053 10.1080/08958370701813273 PMC2442902

[B42] Tewes FJ Meisgen TJ Veltel DJ Roemer E Patskan G Toxicological evaluation of an electrically heated cigarette. Part 3: Genotoxicity and cytotoxicity of mainstream smoke J Appl Toxicol 2003 23 341 348 12975773 10.1002/jat.925

[B43] Terpstra PM Teredesai A Vanscheeuwijck PM Verbeeck J Schepers G Radtke F Kuhl P Gomm W Anskeit E Patskan G Toxicological evaluation of an electrically heated cigarette. Part 4: Subchronic inhalation toxicology J Appl Toxicol 2003 23 349 362 12975774 10.1002/jat.926

[B44] Patskan G Reininghaus W Toxicological evaluation of an electrically heated cigarette. Part 1: Overview of technical concepts and summary of findings J Appl Toxicol 2003 23 323 328 12975771 10.1002/jat.923

[B45] Werley MS Freelin SA Wrenn SE Gerstenberg B Roemer E Schramke H Van Miert E Vanscheeuwijck P Weber S Coggins CR Smoke chemistry, in vitro and in vivo toxicology evaluations of the electrically heated cigarette smoking system series K Regul Toxicol Pharmacol 2008 52 122 139 18590791 10.1016/j.yrtph.2008.05.014

[B46] Stabbert R Voncken P Rustemeier K Haussmann HJ Roemer E Schaffernicht H Patskan G Toxicological evaluation of an electrically heated cigarette. Part 2: Chemical composition of mainstream smoke J Appl Toxicol 2003 23 329 339 12975772 10.1002/jat.924

[B47] Schramke H Meisgen TJ Tewes FJ Gomm W Roemer E The mouse lymphoma thymidine kinase assay for the assessment and comparison of the mutagenic activity of cigarette mainstream smoke particulate phase Toxicology 2006 227 193 210 16963170 10.1016/j.tox.2006.07.019

[B48] Motz GT Eppert BL Wesselkamper SC Flury JL Borchers MT Chronic cigarette smoke exposure generates pathogenic T cells capable of driving COPD-like disease in Rag2-/-mice Am J Respir Crit Care Med 2010 181 1223 1233 926 20133926 10.1164/rccm.200910-1485OC PMC2891493

[B49] Motz GT Eppert BL Sun G Wesselkamper SC Linke MJ Deka R Borchers MT Persistence of lung CD8 T cell oligoclonal expansions upon smoking cessation in a mouse model of cigarette smoke-induced emphysema J Immunol 2008 181 8036 8043 19017996 10.4049/jimmunol.181.11.8036

[B50] Wang H Peng W Weng Y Ying H Li H Xia D Yu W Imbalance of Th17/Treg cells in mice with chronic cigarette smoke exposure Int Immunopharmacol 2012 14 504 512 23044435 10.1016/j.intimp.2012.09.011

[B51] Rinaldi M Maes K De Vleeschauwer S Thomas D Verbeken EK Decramer M Janssens W Gayan-Ramirez GN Long-term nose-only cigarette smoke exposure induces emphysema and mild skeletal muscle dysfunction in mice Dis Model Mech 2012 5 333 341 22279084 10.1242/dmm.008508 PMC3339827

[B52] Gosker HR Langen RC Bracke KR Joos GF Brusselle GG Steele C Ward KA Wouters EF Schols AM Extrapulmonary manifestations of chronic obstructive pulmonary disease in a mouse model of chronic cigarette smoke exposure Am J Respir Cell Mol Biol 2009 40 710 716 18988919 10.1165/rcmb.2008-0312OC

[B53] Toledo AC Magalhaes RM Hizume DC Vieira RP Biselli PJ Moriya HT Mauad T Lopes FD Martins MA Aerobic exercise attenuates pulmonary injury induced by exposure to cigarette smoke Eur Respir J 2012 39 254 264 21700603 10.1183/09031936.00003411

[B54] Motz GT Eppert BL Wortham BW Amos-Kroohs RM Flury JL Wesselkamper SC Borchers MT Chronic cigarette smoke exposure primes NK cell activation in a mouse model of chronic obstructive pulmonary disease J Immunol 2010 184 4460 4469 20228194 10.4049/jimmunol.0903654

[B55] Moriyama C Betsuyaku T Ito Y Hamamura I Hata J Takahashi H Nasuhara Y Nishimura M Aging enhances susceptibility to cigarette smoke-induced inflammation through bronchiolar chemokines Am J Respir Cell Mol Biol 2010 42 304 311 19491340 10.1165/rcmb.2009-0025OC

[B56] Leclerc O Lagente V Planquois JM Berthelier C Artola M Eichholtz T Bertrand CP Schmidlin F Involvement of MMP-12 and phosphodiesterase type 4 in cigarette smoke-induced inflammation in mice Eur Respir J 2006 27 1102 1109 16510458 10.1183/09031936.06.00076905

[B57] Musen MA Gennari JH Wong WW Gardner RM A rational reconstruction of INTERNIST-I using PROTEGE-II Proceedings of the 19th Annual Symposium on Computer Applications in Medical 1995 Philadelphia: Hanley & Belfus, Inc 289 293 PMC2579101 8563287

[B58] Shearer R Motik B Horrocks I HermiT: A Highly-efficient OWL Reasoner5th International Workshop on OWL: Experiences and Directions (OWLED 2008) 2008 Karlsruhe, Germany: Universitaet Karlsruhe 10

[B59] Ogren PV Moore RC, Bilmes JA, Chu-Carroll J, Sanderson M Knowtator: A Protégé Plug-in for Annotated Corpus Construction Proceedings of the 2006 Conference of the North American Chapter of the Association for Computational Linguistics on Human Language Technology: Companion Volume: Demonstrations 2006 New York: ACL 273 275

[B60] Hanisch D Fundel K Mevissen HT Zimmer R Fluck J ProMiner: rule-based protein and gene entity recognition BMC Bioinformatics 2005 6 Suppl 1 S14 15960826 10.1186/1471-2105-6-S1-S14 PMC1869006

[B61] Benkner S Arbona A Berti G Chiarini A Dunlop R Engelbrecht G Frangi AF Friedrich CM Hanser S Hasselmeyer P Hose RD Iavindrasana J Köhler M Iacono LL Lonsdale G Meyer R Moore B Rajasekaran H Summers PE Wöhrer A Wood S @neurIST: infrastructure for advanced disease management through integration of heterogeneous data, computing, and complex processing services IEEE Trans Inf Technol Biomed 2010 14 1365 1377 20435543 10.1109/TITB.2010.2049268

[B62] Stevens R Goble CA Bechhofer S Ontology-based knowledge representation for bioinformatics Briefings Bioinf 2000 1 398 414 10.1093/bib/1.4.398 11465057

[B63] Smith B Ceusters W Klagges B Köhler J Kumar A Lomax J Mungall C Neuhaus F Rector AL Rosse C Relations in biomedical ontologies Genome Biol 2005 6 R46 15892874 10.1186/gb-2005-6-5-r46 PMC1175958

[B64] Musen MA Noy NF Shah NH Whetzel PL Chute CG Story MA Smith B NCBO team The National Center for Biomedical Ontology J Am Med Inform Assoc 2012 19 190 195 22081220 10.1136/amiajnl-2011-000523 PMC3277625

[B65] Gangemi A Catenacci C Ciaramita M Lehmann J Sure Y, Domingu J Modelling Ontology Evaluation and Validation Proceedings of the 2006 European Semantic Web Conference 2006 Berlin: Springer-Verlag 140 154

[B66] Ivchenko O Younesi E Shahid M Wolf A Müller B Hofmann-Apitius M PLIO: an ontology for formal description of protein-ligand interactions Bioinformatics 2011 27 1684 1690 21546398 10.1093/bioinformatics/btr256 PMC3106195

