# Supplementary material for: CSEO – the Cigarette Smoke Exposure Ontology
Source: J Biomed Semantics. 2014 Jul 10;5:31. doi: 10.1186/2041-1480-5-31 (PMC4120729; doi:10.1186/2041-1480-5-31)
Supplement: Additional file 2 — CSEO ontology metrics. [file 2041-1480-5-31-S2.doc]

Additional file 2: Table S2. CSEO ontology metrics

| **Ontology metrices** | **Value** |
| --- | --- |
| Number of classes (without owl:Thing) | 20028 |
| Number of leaves | 14356 |
| Max width/breadth | 2504 |
| Avg. width/breadth | 953.72 |
| Max depth | 21 |
| Total no. of children | 26301 |
| Avg. number of children | 1.32 |
| Avg. depth (avg. root-to-leaf distance) | 11.32 |
| Depth variance (var(d) = E[d^2]-E[d]^2) | 7.76 |
| Width/breadth variance (var(w) = E[w^2]-E[w]^2) | 894681.06 |
| Tangledness (no. nodes with 2+ parents / total no. nodes) | 0.27 |
| Fanout factor (no. leaf classes / number classes) | 0.71 |
