# Supplementary material for: CSEO – the Cigarette Smoke Exposure Ontology
Source: J Biomed Semantics. 2014 Jul 10;5:31. doi: 10.1186/2041-1480-5-31 (PMC4120729; doi:10.1186/2041-1480-5-31)
Supplement: Additional file 3 — Titles of retrieved PubMed abstracts for answering competency questions in Table 1. [file 2041-1480-5-31-S3.doc]

Additional file 3: Table S3. Titles of relevant and irrelevant publications that were retrieved for answering competency questions.

| **Competency Question** | **Query used to search literature** | **Titles of relevant publications** | **Titles of irrelevant publications** |
| --- | --- | --- | --- |
| What are the potential effects of the toxicity induced by tobacco smoke constituents on smokers? | (([CSEO:"Smoke Constituent"]) AND [CSEO:"Toxicity"]) AND [CSEO:"Tobacco"] | - Screening for developmental toxicity of tobacco smoke constituents. - Effects of tobacco and tobacco smoke constituents on cell multiplication in vitro. - Monitoring the tobacco use epidemic II: The agent: Current and emerging tobacco products. - A comprehensive evaluation of the toxicology of cigarette ingredients: aliphatic and aromatic carboxylic acids. - The role of skin painting in predicting lung cancer. - Effects of tobacco smoke constituents on MPTP-induced toxicity and monoamine oxidase activity in the mouse brain. - Toxicological evaluation of honey as an ingredient added to cigarette tobacco. - An analysis of the role of tobacco-specific nitrosamines in the carcinogenicity of tobacco smoke. - Chemical composition, cytotoxicity and mutagenicity of smoke from US commercial and reference cigarettes smoked under two sets of machine smoking conditions. - Evaluation of the potential effects of ingredients added to cigarettes. Part 2: chemical composition of mainstream smoke. - In vitro assessment of reproductive toxicity of tobacco smoke and its constituents. - The generation of formaldehyde in cigarettes--Overview and recent experiments. - A comprehensive evaluation of the toxicology of cigarette ingredients: aromatic and aliphatic alcohol compounds. - A comprehensive evaluation of the toxicology of cigarette ingredients: aliphatic and aromatic carboxylic acids. - A comprehensive evaluation of the toxicology of cigarette ingredients: aliphatic carbonyl compounds. - An overview of the effects of tobacco ingredients on smoke chemistry and toxicity. - Reduced toxicological activity of cigarette smoke by the addition of ammonia magnesium phosphate to the paper of an electrically heated cigarette: subchronic inhalation toxicology. | - Inhibition of neovascularization by environmental agents. - Safety and efficacy of weekly oral oltipraz in chronic smokers. - Exposure to ethanol and tobacco smoke in relation to level of PCNA antigen expression in pancreatic and hepatic rat cells. - The changing cigarette, 1950-1995. |
| Which toxicological studies measure total particulate matter in electrically heated cigarettes are available? | ([CSEO:"Electrically heated cigarette"]) AND [CSEO:"Total Particulate Matter"] | - Toxicological evaluation of an electrically heated cigarette. Part 4: Subchronic inhalation toxicology. - Toxicological evaluation of an electrically heated cigarette. Part 3: Genotoxicity and cytotoxicity of mainstream smoke. - Chemical composition, cytotoxicity and mutagenicity of smoke from US commercial and reference cigarettes smoked under two sets of machine smoking conditions. - Toxicological evaluation of an electrically heated cigarette. Part 1: Overview of technical concepts and summary of findings. - Smoke chemistry, in vitro and in vivo toxicology evaluations of the electrically heated cigarette smoking system series - Toxicological evaluation of an electrically heated cigarette. Part 2: Chemical composition of mainstream smoke. - The mouse lymphoma thymidine kinase assay for the assessment and comparison of the mutagenic activity of cigarette mainstream smoke particulate phase. | N/A |
| Which documents report on the use of experimental mouse models for investigating the effect of cigarette smoke exposure on the risk of COPD? |  | - Chronic cigarette smoke exposure generates pathogenic T cells capable of driving COPD-like disease in Rag2-/- mice. - Persistence of lung CD8 T cell oligoclonal expansions upon smoking cessation in a mouse model of cigarette smoke-induced emphysema. - Imbalance of Th17/Treg cells in mice with chronic cigarette smoke exposure. - Long-term nose-only cigarette smoke exposure induces emphysema and mild skeletal muscle dysfunction in mice. - Aerobic exercise attenuates pulmonary injury induced by exposure to cigarette smoke. - Extrapulmonary manifestations of chronic obstructive pulmonary disease in a mouse model of chronic cigarette smoke exposure. - Chronic cigarette smoke exposure primes NK cell activation in a mouse model of chronic obstructive pulmonary disease. - Aging enhances susceptibility to cigarette smoke-induced inflammation through bronchiolar chemokines. - Involvement of MMP-12 and phosphodiesterase type 4 in cigarette smoke-induced inflammation in mice. | N/A |
