# Supplementary material for: CSEO – the Cigarette Smoke Exposure Ontology
Source: J Biomed Semantics. 2014 Jul 10;5:31. doi: 10.1186/2041-1480-5-31 (PMC4120729; doi:10.1186/2041-1480-5-31)
Supplement: Additional file 4 — Resources used for construction of CSEO. [file 2041-1480-5-31-S4.doc]

Additional file 4: Table S1. Resources used for construction of CSEO

| **Type of resource** | **Subject** |
| --- | --- |
| **Hand book** | - Cigarette Smoke Toxicity: Linking Individual Chemicals to Human Diseases by David Bernhard 2011 |
| **Abstracts** | - Philip Morris publications - CORESTA conference abstracts - Pubmed abstracts related to high-throughput analyses of cigarette smoke exposure experiments - Society of Toxicology (SOT) conference abstracts - Tobacco Science Research Conference (TSRC) abstracts - Various abstracts from toxicological service labs |
| **Dictionaries** | - LIMS - Philip Morris internal databases including instruments and various abbreviation lists |
| **Public ontologies** | - CDISC vocabularies (including SEND) - MGED Ontology - NCBITaxon - NCIt - EFO - ArrayExpress - Anatomical Entity Ontology (AEO) - Basic Vertebrate Anatomy (basic-vertebrate-gross-anatomy) - BioAssay Ontology (BAO) - Bioinformatics operations, types of data, data formats and topics (EDAM) - Biological imaging methods (FBbi) - Biomedical Resource Ontology (BRO) - BioModels Ontology (BioModels) - BioPAX (BP) - BioPortal Metadata (BPMetadata) - Cancer Research and Management ACGT Master Ontology (ACGT) - Cell Culture Ontology (CCONT) - Cell Cycle Ontology (CCO) - Cell Line Ontology (CLO) - Cell line ontology (MCCL) - Clinical Measurement Ontology (CMO) - Common Terminology Criteria for Adverse Events (CTCAE) - Environment Ontology (ENVO) - Experimental Conditions Ontology (XCO) - FDA Medical Devices (2010) (FDA-MedDevice) - HUGO (HUGO) - Human disease ontology (DOID) - ICD10 (ICD10) - International Classification of Diseases (ICD9CM) - Mass spectrometry (MS) - Measurement Method Ontology (MMO) - MedDRA (MDR) - Medical Subject Headings (MeSH) (MSH) - Mouse gross anatomy and development (EMAP) - Protein modification (MOD) - Proteomics data and process provenance (ProPreO) - Quantities, Units, Dimensions and Types (QUDT) - Sample processing and separation techniques (SEP) - Smoking Behavior Risk Ontology (SBRO) - SNOMED Clinical Terms (SNOMEDCT) - Systems Biology (SBO) - Tissue Microarray Ontology (TMA) - Units of measurement (UO) - Opentoxipedia |
